# Modulation of γ-secretase by EVP-0015962 reduces amyloid deposition and behavioral deficits in Tg2576 mice

**DOI:** 10.1186/1750-1326-7-61

**Published:** 2012-12-18

**Authors:** Kathryn Rogers, Kevin M Felsenstein, Lori Hrdlicka, Zhiming Tu, Faris Albayya, Winnie Lee, Sarah Hopp, Mary-Jo Miller, Darcie Spaulding, Zhiyong Yang, Hilliary Hodgdon, Scott Nolan, Melody Wen, Don Costa, Jean-Francois Blain, Emily Freeman, Bart De Strooper, Veerle Vulsteke, Louise Scrocchi, Henrik Zetterberg, Erik Portelius, Birgit Hutter-Paier, Daniel Havas, Michael Ahlijanian, Dorothy Flood, Liza Leventhal, Gideon Shapiro, Holger Patzke, Richard Chesworth, Gerhard Koenig

**Affiliations:** 1 EnVivo Pharmaceuticals, Inc, 500 Arsenal Street, Watertown, MA, 02472, USA; 2 VIB Center for The Biology of Disease, Herestraat 49, Box 602, Leuven, 3000, Belgium; 3 KULeuven and University Hospitals, Center for Human Genetics and LIND, Leuven, 3000, Belgium; 4 Amorfix Life Sciences, 3403 American Drive, Mississauga, Ontario, L4V 1T4, Canada; 5 Clinical Neurochemistry Laboratory, Department of Psychiatry and Neurochemistry, Institute of Neuroscience and Physiology, The Sahlgrenska Academy, University of Gothenburg, Mölndal, S-43180, Sweden; 6 UCL Institute of Neurology, Queen Square, London, WC1N 3BG, UK; 7 QPS Austria GmbH, Parkring 12, Grambach, A-8074, Austria; 8 Present address: CoMentis Research Facility, 865 Research Parkway, Suite 400, Oklahoma City, OK, 73104, USA; 9 Present address: Department of Neuroscience, Center for Translational Research in Neurodegenerative Disease, University of Florida College of Medicine, 1275 Center Drive, BMS J-484, Gainesville, FL 32610, USA; 10 Present address: Eisai, Inc., 4 Corporate Dr, Andover, MA, 01810, USA; 11 Present address: Neuroscience Department, The Ohio State University, Columbus, OH, 43210, USA; 12 Present address: Galenea, 300 Technology Square # 2, Cambridge, MA, 02139, USA; 13 Present address: University of Maryland Baltimore, 650 West Baltimore St., Room 7211, Baltimore, MD, 21201, USA; 14 Present address: Biopharmaceuticals, Apotex, Inc, 150 Signet Drive, Toronto, Ontario, M9L 1T9, Canada; 15 Present address: Neuroscience Biology, Bristol-Myers Squibb, Research and Development, 5 Research Parkway, Wallingford, CT, 06492, USA; 16 Present address: Pharmore, Inc, 5507 NW 80th Ave, Gainesville, FL, 32653, USA; 17 Present address: Epizyme, Inc, 325 Vassar Street, Suite 2B, Cambridge, MA, 02139, USA

**Keywords:** γ-secretase, Modulation, NSAID, Cognition, Amyloid, Alzheimer’s disease

## Abstract

**Background:**

A hallmark of Alzheimer’s disease is the presence of senile plaques in human brain primarily containing the amyloid peptides Aβ_42_ and Aβ_40_. Many drug discovery efforts have focused on decreasing the production of Aβ_42_ through γ-secretase inhibition. However, identification of γ-secretase inhibitors has also uncovered mechanism-based side effects. One approach to circumvent these side effects has been modulation of γ-secretase to shift Aβ production to favor shorter, less amyloidogenic peptides than Aβ_42_, without affecting the overall cleavage efficiency of the enzyme. This approach, frequently called γ-secretase modulation, appears more promising and has lead to the development of new therapeutic candidates for disease modification in Alzheimer’s disease.

**Results:**

Here we describe EVP-0015962, a novel small molecule γ-secretase modulator. EVP-0015962 decreased Aβ_42_ in H4 cells (IC_50_ = 67 nM) and increased the shorter Aβ_38_ by 1.7 fold at the IC_50_ for lowering of Aβ_42_. Aβ_Total_, as well as other carboxyl-terminal fragments of amyloid precursor protein, were not changed. EVP-0015962 did not cause the accumulation of other γ-secretase substrates, such as the Notch and ephrin A4 receptors, whereas a γ-secretase inhibitor reduced processing of both. A single oral dose of EVP-0015962 (30 mg/kg) decreased Aβ_42_ and did not alter Aβ_Total_ peptide levels in a dose-dependent manner in Tg2576 mouse brain at an age when overt Aβ deposition was not present. In Tg2576 mice, chronic treatment with EVP-0015962 (20 or 60 mg/kg/day in a food formulation) reduced Aβ aggregates, amyloid plaques, inflammatory markers, and cognitive deficits.

**Conclusions:**

EVP-0015962 is orally bioavailable, detected in brain, and a potent, selective γ-secretase modulator *in vitro* and *in vivo*. Chronic treatment with EVP-0015962 was well tolerated in mice and lowered the production of Aβ_42_, attenuated memory deficits, and reduced Aβ plaque formation and inflammation in Tg2576 transgenic animals. In summary, these data suggest that γ-secretase modulation with EVP-0015962 represents a viable therapeutic alternative for disease modification in Alzheimer’s disease.

## Background

Alzheimer’s disease (AD) is a progressive neurodegenerative disease, first described in 1907 [1]. Functional changes in this devastating disease include early memory deficits and later deficits affecting language, executive functioning, perception, and/or execution of complex motor patterns [2]. The neuropathology in AD is characterized by the presence of extracellular amyloid (Aβ) plaques and intracellular neurofibrillary tangles in the cerebral cortex, hippocampus, and amygdala, as well as other brain regions associated with memory and other domains of cognition [3]. The major constituents of the amyloid plaques are well established and include the 4 kDa Aβ peptides, primarily Aβ_42_ and Aβ_40_[4-7]. There is considerable evidence suggesting that Aβ_42_ is deposited early in the amyloid plaques and forms the seed for later deposition of other Aβ species [5,8]. This evidence has suggested that an early and constant reduction in Aβ_42_ in prodromal AD might delay the onset or slow the progression of the disease by affecting the rate of plaque formation. Aβ_42_ is derived from sequential processing of amyloid precursor protein (APP) by two proteases, β-site APP-cleaving enzyme (BACE) and γ-secretase [9,10]. Initially, BACE cleaves APP in its ectodomain, followed by γ-secretase cleavage in the APP transmembrane domain [9,10], which ultimately produces a number of Aβ peptides of various lengths [11]. Significantly, most familial AD mutations in the APP gene are found around the BACE or γ-secretase cleavage sites, and thus both BACE and γ-secretase have been targets for therapeutic intervention in AD [8,12].

γ-Secretase is a large complex composed of the four polypeptides: presenilin (PS-1 or PS-2), nicastrin (Nct), presenilin enhancer 2 (Pen-2), and anterior pharynx-defective 1 (Aph-1) [7]. γ-Secretase is responsible for the processing of more than 70 transmembrane proteins involved in normal cellular processes, including regulation of cell fate, cell adhesion, migration, neurite outgrowth, synaptogenesis, calcium homeostasis, transport of membrane proteins, and cell signaling [13,14]. Mutations in PS, the putative catalytic site of the γ-secretase complex, and in APP around the γ-secretase cleavage site, were shown to increase the ratio of Aβ_42_/Aβ_40_, strengthening support for γ-secretase inhibition for disease modification in AD [8,12]. While inhibition of γ-secretase would produce the desired Aβ reduction, it would also affect the proteolysis of its other substrates. The Notch receptor is one of these substrates [15], which is of particular interest since the inhibition of its proteolytic processing by γ-secretase inhibitors (GSIs) has been shown to result in the suppression of intestinal goblet cell differentiation and in immunosuppression [16]. Several GSIs have entered clinical trials in AD, but unfortunately, have produced toxicities that are presumably mechanism-based. In particular, one compound (Semegacestat) produced drug-related rashes, lightening of hair color, skin cancer, and more importantly, worsening of cognition and the ability to perform activities of daily living [17-19]. These mechanism-based toxicities of GSIs have been attributed to the inhibition of Notch receptor processing and to the accumulation of the APP β-carboxyl-terminal fragment (CTF) [16,19].

Neuroinflammation is another pathological hallmark of AD and is characterized by the presence of activated microglia and reactive astrocytes surrounding the amyloid plaques [20]. The question of whether the gliosis is causative or a compensatory result of the amyloid plaque deposition has been the subject of ongoing discussions and studies since it was first described [20,21]. For example, numerous retrospective studies associated a lower incidence of AD in patient populations that were prescribed non-steroidal anti-inflammatory drugs (NSAIDs) for other conditions [20]. It was therefore assumed that the NSAID therapy exerted positive effects on AD by reducing neurotoxic inflammation through the reduction of cyclooxygenase (COX) activities [20]. However, Weggen et al. [22,23] described a series of *in vitro* and *in vivo* studies utilizing several NSAIDs that produced a preferential reduction of Aβ_42_ compared to Aβ_40_. This reduction of Aβ_42_ was accompanied by a concomitant increase in Aβ_38_, a shorter, less amyloidogenic Aβ peptide [12], rather than the inhibition of all carboxyl-terminal processing of APP [22,23]. Furthermore, they demonstrated that the effects of NSAIDs on the preferential reduction of Aβ_42_ peptide levels were not linked to the inhibition of COX or other enzymes, but rather to a specific action on γ-secretase [22,24]. The shift in production of Aβ peptides from the longer, toxic forms to the shorter, less toxic forms by NSAIDs has been termed γ-secretase modulation. This has sparked a flurry of activity directed at the development of compounds that modulate APP cleavage by γ-secretase and that could avoid the toxicities arising from the complete enzymatic inhibition of γ-secretase. Several recent publications have described second generation γ-secretase modulators (GSMs) [25-29] and Notch-sparing GSIs [18,30]. Here we present the *in vitro* and *in vivo* characterization of EVP-0015962, a potent, second generation GSM that specifically modulated production of Aβ_42_ and Aβ_38_ without affecting other γ-secretase substrates. In transgenic mice over-expressing APP, EVP-0015962 was well tolerated following chronic dosing, produced reductions in amyloid plaque burden and neuroinflammation, and improved cognition.

## Results

### EVP-0015962 selectively reduces the levels of Aβ_42 _*in vitro*

**Figure 1:**
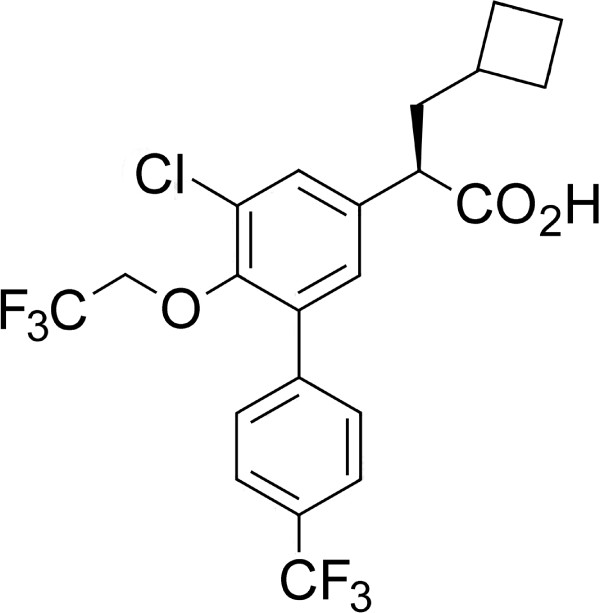
The chemical structure of EVP-0015962.

**Figure 2:**
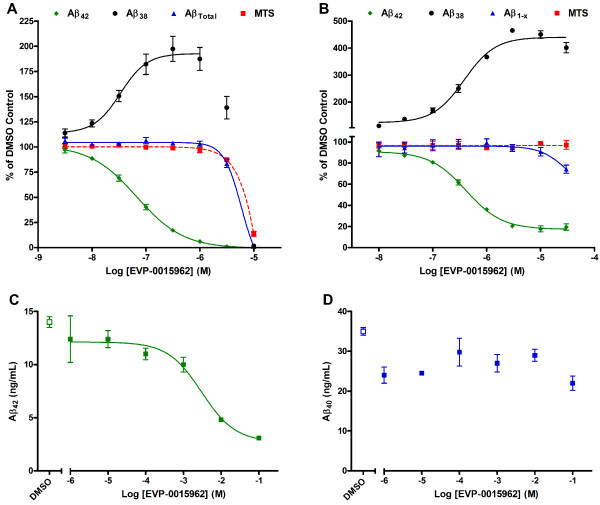
**EVP-0015962 is a potent γ-secretase modulator. A.** Concentration response curves for Aβ_42_, Aβ_38_, and Aβ_Total_ determined by enzyme-linked immunosorbent assay (ELISA), and for cell viability in the H4-APP_751_ cell assay. IC_50_ = 67 nM for Aβ_42_ and EC_50_ = 33 nM for Aβ_38_. For cell viability determined using the MTS assay, IC_50_ = 5.56 μM. IC_50_ > 3 μM for Aβ_Total_. For Aβ_38_, the EC_50_ value is determined for the concentration range of 3 nM to 1 μM of EVP-0015962, to avoid the portion of the curve where toxicity is observed. N = 6 independent experiments. **B.** Concentration response curves for Aβ_42_, Aβ_38_, and Aβ_1-x_ determined by ELISA in media and for MTS from rat primary neocortical cultures. IC_50_ = 427 nM for Aβ_42_ and EC_50_ = 384 nM for Aβ_38_. IC_50_ > 30 μM for Aβ_1-x_ and the MTS assay. N = 4 independent experiments for Aβ_42_, Aβ_1-x_, and MTS and 2 for Aβ_38_. **C**. Concentration response curve for Aβ_42_ in the cell-free assay. IC_50_ = 3.9 μM. **D**. Concentration response curve for Aβ_40_ in the cell-free assay. In the cell-free assays, Aβ was determined by AlphaLisa in triplicate. All data are expressed as mean ± standard error of the mean (SEM). For some data points, error bars are smaller than the sizes of the data points.

**Figure 3:**
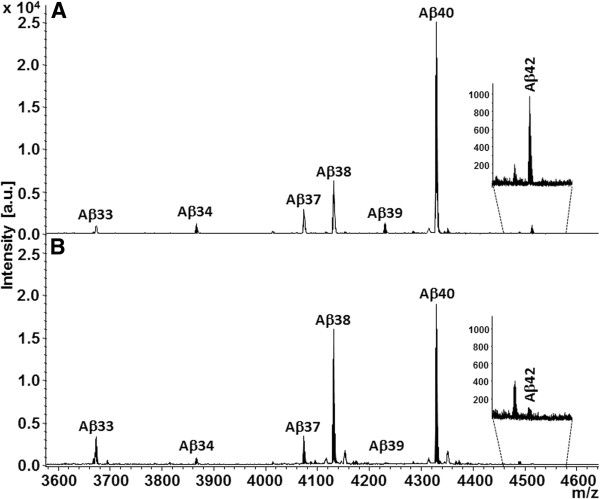
**EVP-0015962 selectively affects processing of Aβ isoforms. A** and **B**. Effect of EVP-0015962 treatment on production of Aβ isoforms, measured by MALDI-TOF mass spectrometry. Mass spectra from H4-APP_751_ cells treated with DMSO (**A**) and 700 nM EVP-0015962 (**B**). It should be noted that a relative quantification cannot be interpreted as a direct reflection of an absolute or relative abundance of a species since the ionization efficiency and hydrophobicity might be different for different Aβ isoforms. N = 1 experiment.

In the course of a traditional drug discovery effort aimed at identifying novel compounds with GSM activity, EVP-0015962, (*R*)-2-(5-chloro-6-(2,2,2-trifluoroethoxy)-4’-(trifluoromethyl)biphenyl-3-yl)-3-cyclobutylpropanoic acid, was identified and characterized (Figure 1). Using human neuroglioma H4 cells, stably transfected with human wild type APP_751_ (H4-APP_751_ cells), EVP-0015962 demonstrated a dose-dependent decrease in the levels of Aβ_42_ after overnight incubation, while the levels of Aβ_Total_ remained unchanged up to 3 μM (Figure 2A). The mean IC_50_ for Aβ_42_ was 67 ± 5 nM (n = 6). No effect was observed on Aβ_Total_ until the concentration of EVP-0015962 reached levels at which cytotoxicity was observed in the MTS assay (IC_50_ = 5.56 ± 0.51 μM, n = 6). The mean EC_50_ for Aβ_38_ was 33 ± 4 nM (n = 6). At the IC_50_ for Aβ_42_, the mean fold increase in Aβ_38_ was 1.7. The patterns of the Aβ isoforms were also analyzed by matrix-assisted laser desorption/ionization-time-of-flight (MALDI-TOF) mass spectrometry after treatment of H4-APP_751_ cells with DMSO or 700 nM (IC_90_) of EVP-0015962 (Figure 3A-B). The relative quantification of selected Aβ isoform peak heights for EVP-0015962 treatment compared with DMSO treatment showed that EVP-0015962 decreased Aβ_42_ and Aβ_39_ production, increased Aβ_38_ and Aβ_33_ production, and did not change Aβ_40_ and Aβ_37_ production.

The potency of EVP-0015962 was also evaluated in rat primary neocortical cultures. In these studies, EVP-0015962 lowered the levels of Aβ_42_ with an average IC_50_ of 427 ± 52 nM (n = 4), while the IC_50_ values for the levels of Aβ_1-x_ peptides and cytotoxicity were > 30 μM (n = 4) (Figure 2B). The mean EC_50_ for Aβ_38_ was 384 ± 11 nM (n = 2). At the IC_50_ for Aβ_42_, the mean fold increase in Aβ_38_ was 3.0. These results suggest that EVP-0015962 acts as a typical GSM.

### EVP-0015962 does not impair other γ-secretase cleavages

**Figure 4:**
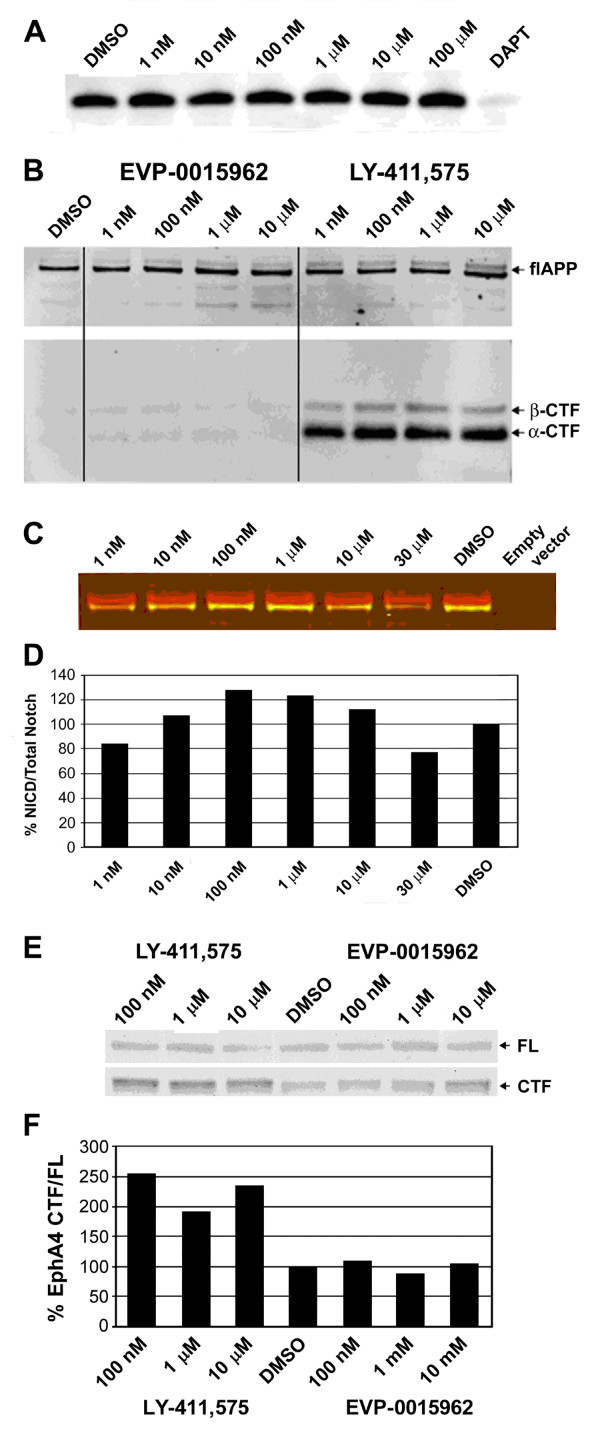
**EVP-0015962 does not alter processing of APP or other γ-secretase substrates. A.** Effect of EVP-0015962 treatment on the ε-cleavage of APP β-CTF, measured in a cell-free assay with Aph1A_L_ containing γ-secretase complexes. Representative Western blot of AICD fragments, for EVP-0015962 (1 nM to 100 μM) and DAPT (10 μM). DMSO is the control lane. N = 2 experiments. **B.** Full length APP (flAPP), α-CTF, and β-CTF from H4-APP_751_ cell lysates, analyzed by Western blot after a 16-h treatment with 1 nM to 10 μM of LY-411,575 or EVP-0015962. N = 1 experiment. **C** and **D.** Effect of EVP-0015962 treatment (1 nM to 30 μM) on the S3 cleavage of NotchΔE to form NICD, measured in transfected HEK293 cells. **C.** Representative Western blot of NICD fragments fluorescently labeled with cleaved Notch1 antibody (green) and Myc-tagged antibody (red). The NICD fragments are detected as yellow in an overlay of both antibodies. DMSO is the control lane and Empty vector is a lysate from cells without the NotchΔE construct. **D.** NICD intensity histogram corresponding to the Western blot in E. NICD is quantified as a percentage of total Notch. N = 2 experiments. **E** and **F.** Effect of 24 h of treatment with 100 nM to 10 μM of LY-411,575 or EVP-0015962 on the production of EphA4 CTF in cell lysates from rat primary neocortical cultures. **E.** Western blot of full length (FL) EphA4 (upper band) and EphA4 CTF (lower band). **F.** Ratio of EphA4 CTF to full length EphA4 corresponding to the Western blot in E. N = 1 experiment.

Most, if not all, γ-secretase substrates undergo ectodomain shedding; and the resulting CTFs are released from the membrane by γ-secretase [14]. This cleavage also leads to the production of intracellular domains (ICDs) of the γ-secretase substrates [14]. Inhibition or loss of γ-secretase has been shown to prevent the formation of these ICDs, and consequently to lead to the accumulation of CTFs of the γ-secretase substrates [15,19,29-33]. In the case of APP, the products of α- and β-secretase cleavages (α- and β-CTFs, respectively) are cleaved by γ-secretase to produce the APP intracellular domain (AICD). As shown in Figure 4A, in a cell-free system using reconstituted γ-secretase, up to 100 μM of EVP-0015962 did not prevent the production of AICD from APP β-CTF, whereas treatment with the GSI, N-(N-(3,5-difluorophenacetyl)-L-alanyl)-S-phenylglycine t-butyl ester (DAPT), almost completely abolished AICD production at 10 μM. In this cell-free assay, EVP-0015962 selectively reduced Aβ_42_ levels with an IC_50_ of 3.9 μM, without affecting the levels of Aβ_40_ (Figure 2C-D). Recently, accumulation of APP β-CTF after chronic treatment with GSIs has been implicated in cognitive impairment [19]. We also showed that EVP-0015962 treatment of H4-APP_751_ cells did not cause the accumulation of APP α- or β-CTFs at concentrations up to 10 μM, whereas the GSI LY-411,575 [16] led to their accumulation at a concentration as low as 1 nM (Figure 4B). Full length APP was not altered by treatment with either EVP-0015962 or LY-411,575, consistent with published findings [27,30,33,34].

The inhibition of Notch intracellular domain (NICD) production is a well-characterized consequence of γ-secretase inhibition and has been linked to many of the toxicities associated with GSIs [16-18,31]. In HEK293 cells expressing NotchΔE, EVP-0015962 did not affect levels of NICD produced or cell viability at concentrations up to 30 μM (Figure 4C-D). Finally, inhibition of the processing of one member of the ephrin (Eph) receptor family (EphA4) by GSIs was reported to inhibit dendritic spine formation in primary neurons [35]. The effects of EVP-0015962 and LY-411,175 on EphA4 CTF accumulation in rat primary neocortical cultures were assessed. Up to 10 μM of EVP-0015962 did not result in EphA4 CTF accumulation, whereas a concentration as low as 100 nM of LY-411,175 led to EphA4 CTF accumulation (Figure 4E-F).

### Single doses of EVP-0015962 reduce Aβ_42_ in Tg2576 mice

**Figure 5:**
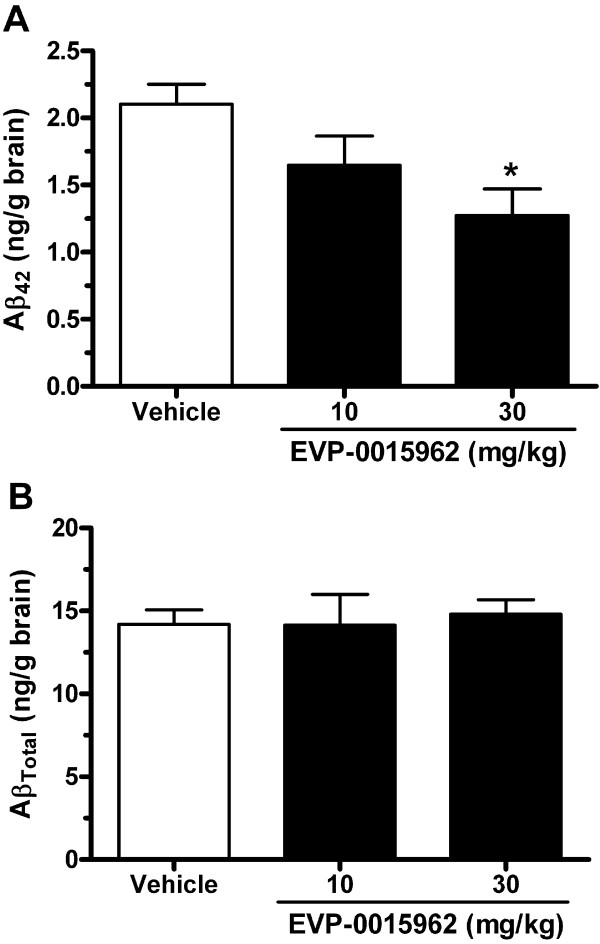
**In Tg2576 mice, a single dose of EVP-0015962 decreases brain Aβ**_**42 **_**without altering Aβ**_**Total**_**.** Brain concentrations of Aβ_42_ (**A**) and Aβ_Total_ (**B**), measured 4 h after 10 or 30 mg/kg, p.o. of EVP-0015962 by sandwich ELISAs, following TBS extraction. Graphs illustrate mean + SEM, N = 4 per group. Difference from the vehicle-treated group: *p < 0.05 (1-way ANOVA, *post-hoc* Newman-Keuls test).

Acute dosing of EVP-0015962 was studied in male Tg2576 mice at 21 weeks, an age before the onset of Aβ deposition in this transgenic APP over-expression model [36]. Four hours after administration of EVP-0015962 at 10 or 30 mg/kg, p.o., Aβ peptides were extracted from brain using Tris buffered saline (TBS). Overall, Aβ_42_ was significantly decreased by EVP-0015962 (F[2,9] = 4.74, p = 0.039, Figure 5A). The decreases compared to vehicle-treated mice were 22% for 10 mg/kg (NS) and 39% for 30 mg/kg of EVP-0015962 (p < 0.05). Aβ_Total_ remained unchanged by EVP-0015962 treatment (F[2,9] = 0.085, p = NS, Figure 5B).

### Chronic administration of EVP-0015962 is well tolerated and decreases brain Aβ levels in Tg2576 mice

The goal of the chronic study was to determine the effects of EVP-0015962 on AD-like pathology in Tg2576 mice when daily treatment was begun at 17–26 weeks of age, before visible Aβ deposition is detected in this mouse model [36]. EVP-0015962 was administered to Tg2576 mice for 50 weeks in a food formulation. The concentrations of EVP-0015962 used in the food formulations were determined in pilot studies. Based on the minimum effective brain exposures to produce Aβ reductions after single doses of 10 and 30 mg/kg, p.o. in Tg2576 mice (1.3 and 4.3 μM, respectively at 4 h post-dosing), the compound concentrations of 20 and 60 mg/kg/day in the food formulations were selected. In C57BL/6 mice fed with the food formulations of 20 and 60 mg/kg/day for 3 or 7 days, the ranges of brain concentrations were 1.5–3.1 and 4.9–9.7 μM, respectively (n = 3 studies). After 50 weeks, brain concentrations of EVP-0015962 in the chronically treated Tg2576 mice were 2.5 ± 0.2 and 8.3 ± 0.6 μM for 20 and 60 mg/kg/day, respectively. Thus, the brain concentrations of EVP-0015962 were within the expected range observed after short-term treatments and did not demonstrate accumulation or cytochrome P450 enzyme induction.

**Figure 6:**
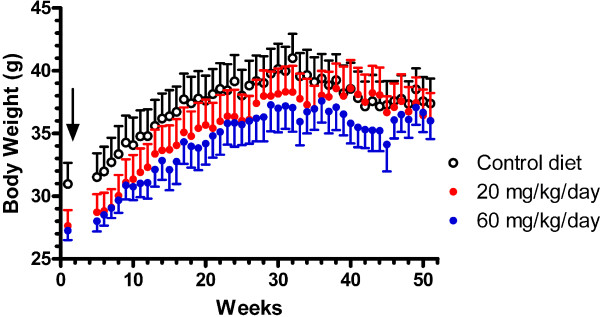
**Chronic daily exposure to EVP-0015962 in Tg2576 mice is well tolerated.** Body weight change in Tg2576 mice, measured weekly beginning the week prior to chronic dosing with EVP-0015962 in a food formulation at 20 or 60 mg/kg/day and continuing through the course of treatment. There are no group differences in body weight change (2-way repeated measures ANOVA, within subjects variable – week and between subjects variable – drug treatment). Data are the mean ± SEM of 6–8 mice per group. Arrow indicates the start of treatment with EVP-0015962.

Body weights were tracked throughout the study to assess the long term tolerability of EVP-0015962 (Figure 6). All mice gained a significant amount of weight during the course of treatment (p < 0.001), and there were no significant differences in body weight among the treatment groups at any time point during the study. These data indicated that chronic EVP-0015962 treatment was well tolerated.

**Figure 7:**
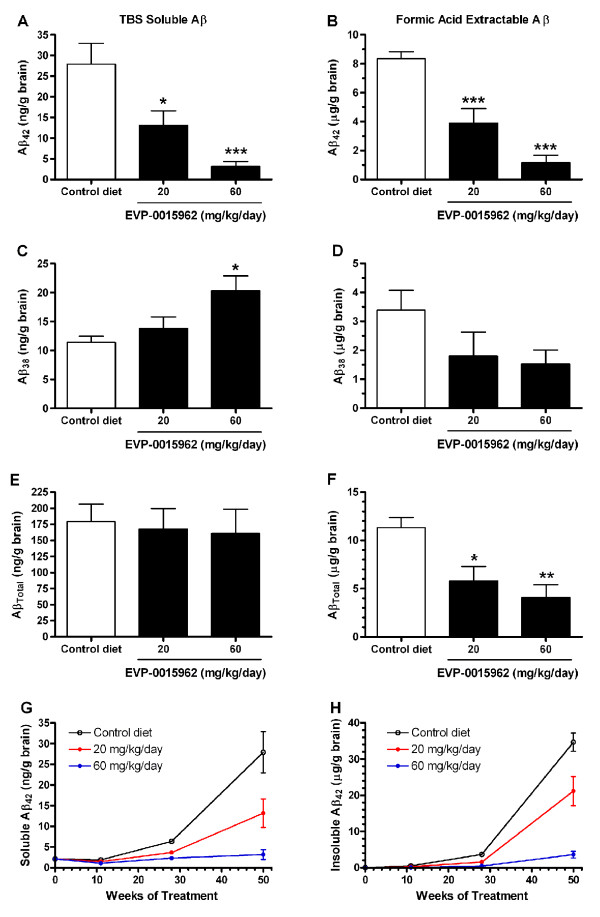
**Chronic daily exposure to EVP-0015962 in Tg2576 mice reduces Aβ peptide levels. A-F.** TBS soluble (**A, C, E**) and formic acid extractable (**B, D, F**) Aβ peptide levels determined by ELISA from the brains of Tg2576 mice after 50 weeks of treatment with EVP-0015962 for Aβ_42_ (**A-B**), Aβ_38_ (**C-D**), and Aβ_Total_ (**E-F**). N = 5 mice per group. Differences from the control diet group: *p < 0.05, **p < 0.01, ***p < 0.001 (1-way ANOVA, *post-hoc* Newman-Keuls test). **G-H.** Aβ_42_ peptide levels measured at the beginning of treatment and after 11, 28, and 50 weeks of treatment with EVP-0015962, showing the time course of accumulation of Aβ_42_. **G.** TBS soluble Aβ_42_. **H.** Insoluble Aβ_42_. At 11 and 28 weeks, insoluble Aβ_42_ was extracted with only formic acid. At 0 and 50 weeks, the 4-step procedure was used, and the insoluble fraction is the sum of the Aβ_42_ extracted with Triton®-X, SDS, and formic acid. N = 4–6 mice per group and time point. Graphs illustrate mean ± SEM.

The effects of chronic EVP-0015962 treatment on sequentially extracted Aβ using TBS, Triton®-X, sodium dodecyl sulfate (SDS), and formic acid [37] were also evaluated. The results of the Triton®-X fraction resembled those of the TBS fraction, and the results of the SDS fraction resembled those of the formic acid fraction. Therefore only the TBS (Figure 7A, C, E) and formic acid (Figure 7B, D, F) fractions are described. Aβ_42_ was significantly lowered in both the TBS soluble and formic acid extractable fractions (TBS, F[2,12] = 12.3, p = 0.001; formic acid, F[2,12] = 27.2, p < 0.001; Figure 7A-B). The percent reduction in Aβ_42_ in the TBS soluble fraction was 53% for 20 mg/kg/day (p < 0.05) and 89% for 60 mg/kg/day (p < 0.001). Similarly, formic acid extractable Aβ_42_ was reduced for the 20 and 60 mg/kg/day doses by 53% and 86%, respectively (p < 0.001). In contrast, Aβ_38_ in the TBS soluble fraction was increased by treatment with EVP-0015962 (F[2,12] = 5.37, p = 0.022, Figure 7C). Treatment with 60 mg/kg/day of EVP-0015962 increased Aβ_38_ by 78% (p < 0.05), but 20 mg/kg/day did not produce a significant increase. In the formic acid extractable fraction, Aβ_38_ was not significantly changed, despite a trend (p = 0.055 by t-test) towards a decrease in the 60 mg/kg/day group (Figure 7D). Aβ_Total_ was unchanged in the TBS soluble fraction (Figure 7E), but was reduced in the formic acid extractable fraction (F[2,12] = 8.59, p = 0.005, Figure 7F). The reductions were 49% (p < 0.05) and 64% (p < 0.01) at 20 and 60 mg/kg/day of EVP-0015962, respectively. During the chronic study, Aβ peptide levels were evaluated after 11 and 28 weeks of dosing in additional Tg2576 mice. Aβ_42_ peptide levels from the TBS soluble and insoluble fractions increased exponentially in mice on the control diet (Figure 7G-H). Treatment with 60 mg/kg/day of EVP-0015962 for 50 weeks prevented the dramatic increase in both soluble and insoluble Aβ_42_ that is normally associated with Aβ deposition in this mouse model. The lower dose of EVP-0015962 was partially effective in preventing the increase in Aβ_42_ over the course of the 50-week treatment.

**Figure 8:**
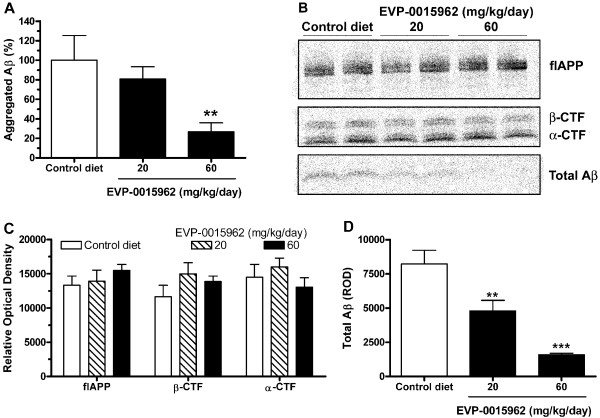
**Chronic daily exposure to EVP-0015962 in Tg2576 mice reduces Aβ without affecting APP α- and β-CTFs. A.** Aggregated Aβ, measured by the A4 assay and expressed as a percent of the control diet group in 17-month-old mice. N = 9–10 mice per group. Difference from the control diet group: **p < 0.01 (Kruskal-Wallis ANOVA, *post-hoc* Dunn’s test). **B.** Representative Western blot for the different peptide species of APP for Tg2576 mice on the control diet and treated with 20 or 60 mg/kg/day of EVP-0015962. **C.** Quantification of relative optical density (ROD) from Western blots for full length APP (flAPP), β-CTF, and α-CTF. **D.** Quantification of ROD from Western blots for Total Aβ. N = 3–4 mice per group. Graphs illustrate mean + SEM. Differences from the control diet group: **p < 0.01, ***p < 0.001 (1-way ANOVA, *post-hoc* Newman-Keuls test).

Aggregated Aβ, measured by the Amorfix aggregated Aβ assay, was detectable in all groups of Tg2576 mice and was reduced by 50 weeks of treatment with EVP-0015962 (H[3] = 10.28, p = 0.006, Figure 8A). There was a significant reduction in aggregated Aβ of 73% in the group receiving 60 mg/kg/day of EVP-0015962 compared to the control diet group (p < 0.01).

Brain samples were also analyzed by Western blotting to determine whether 50 weeks of treatment with EVP-0015962 resulted in accumulation of APP CTFs. There were no increases in the levels of full length APP, β-CTF, and α-CTF (Figure 8B-C) for mice at either dose compared to mice on control diet. On the other hand, total Aβ was significantly and dose dependently decreased by EVP-0015962 treatment (F[2,7] = 27.5, p < 0.001, Figure 8B, D). Compared to Tg2576 mice on the control diet, the reductions were 42% (p < 0.01) and 81% (p < 0.001) for the 20 and 60 mg/kg/day groups, respectively. This sugges-ted that while long-term treatment with EVP-0015962 decreased Aβ levels, it did not inhibit the overall processing of APP CTFs by γ-secretase *in vivo*.

### Chronic treatment with EVP-0015962 inhibits plaque formation and inflammation

**Figure 9:**
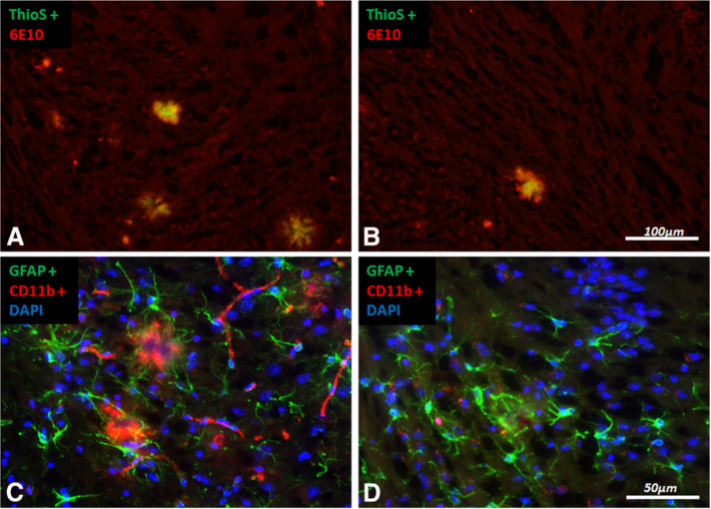
**Chronic daily exposure to EVP-0015962 in Tg2576 mice reduces Aβ deposition and reactive gliosis.** Representative sections illustrating staining of amyloid plaques with ThioflavinS (green) and by immunohistochemistry (6E10, red) in the subiculum of the hippocampus from Tg2576 mice fed either a control diet (**A**) or treated with 20 mg/kg/day of EVP-0015962 (**B**). Representative sections illustrating immunohistochemical staining of reactive astrocytes (GFAP, green) and activated microglia (CD11b, red) in the subiculum from Tg2576 mice, fed either a control diet (**C**) or treated with 60 mg/kg/day of EVP-0015962 (**D**). Nuclei are stained with 4',6-diamidino-2-phenylindole (DAPI, blue). In **C**, amyloid deposits are visualized by the presence of interspersed fine microglial processes and are surrounded by activated microglia and in turn by GFAP-positive astrocytes.

Histological evaluation of amyloid plaques was performed with ThioflavinS fluorescence to visualize compact cores specifically and with 6E10 antibody immunostaining for the compact cores and the surrounding halo of more diffuse Aβ (Table 1, Figure 9A-B). Tg2576 mice treated for 50 weeks with EVP-0015962 showed a significant reduction in percent area and number of amyloid plaques in the hippocampus by immunostaining with 6E10 antibody (percent area, F[2,18] = 4.13, p = 0.033; number, F[2,18] = 4.79, p = 0.022). There was no effect on the mean plaque size. These reductions were seen for both the 20 and 60 mg/kg/day groups (p < 0.05). In the neocortex, the reductions in percent area and number of 6E10-positive amyloid plaques were trends (percent area, F[2,18] = 2.60, p = 0.102; number, F[2,18] = 3.51, p = 0.052). In addition, EVP-0015962 significantly reduced the percent area and number of ThioflavinS-positive amyloid plaque cores in the hippocampus (percent area, F[2,18] = 8.13, p = 0.003; number, F[2,18] = 4.31, p = 0.029). The reductions in the hippocampus reached significance for both the 20 and 60 mg/kg/day groups for percent area (p < 0.01) and number (p < 0.05) of plaque cores. In the neocortex, the percent area and number of ThioflavinS-positive plaque cores were also significantly reduced (percent area, F[2,18] = 5.20, p = 0.017; number, F[2,18] = 3.57, p = 0.049). At the high dose, percent area of plaque cores was significantly reduced in the neocortex (p < 0.05), while plaque number trended lower (t-test, p = 0.054). The size of ThioflavinS-positive plaque cores trended lower in the neocortex and hippocampus after EVP-0015962 treatment (neocortex, F[2,18] = 3.53, p = 0.051; hippocampus, F[2,18] = 2.50, p = 0.110). In the neocortex, the *post hoc* test for plaque core size was nevertheless significant for the comparison of the control diet group with the group treated with 60 mg/kg/day of EVP-0015962 (p < 0.05).

**Table 1:** Mean (± sem) percent of control after chronic of treatment with EVP-0015962 at 20 or 60 mg/kg/day

	**Neocortex**			**Hippocampus**		
**Stain and pathology**	**Control**	**EVP-0015962 (mg/kg/day)**		**Control**	**EVP-0015962 (mg/kg/day)**	
**Parameter**	**diet**	**20**	**60**	**diet**	**20**	**60**
**6E10 immunostaining of Aβ plaque pathology**						
Percent area	100 (10.8)	69.2 (9.7)	65.3 (16.3)	100 (10.8)	62.1 (13.2)*	53.1 (15.2)*
Number/mm^2^	100 (7.9)	65.2 (8.8)	71.3 (15.0)	100 (9.7)	61.0 (12.2)*	53.9 (16.8)*
Size (μm^2^)	100 (6.5)	105.5 (8.8)	88.5 (5.4)	100 (16.4)	99.4 (15.7)	104.1 (11.6)
**ThioflavinS staining of Aβ plaque pathology**						
Percent area	100 (18.0)	64.3 (14.3)	29.9 (7.3)*	100 (14.7)	40.7 (16.9)**	27.3 (6.5)**
Number/mm^2^	100 (12.4)	64.3 (8.0)	59.6 (14.1)	100 (12.7)	55.2 (15.6)*	50.7 (12.8)*
Size (μm^2^)	100 (8.5)	87.8 (12.2)	58.1 (10.6)*	100 (6.8)	56.5 (16.3)	58.9 (27.0)
**GFAP immunostaining of reactive astrocytes**						
Percent area	100 (12.5)	95.8 (14.5)	61.6 (7.6)	100 (9.0)	95.8 (7.7)	87.2 (9.7)
**CD11b immunostaining of activated microglia**						
Percent area	100 (12.8)	86.3 (5.0)	66.2 (7.7)	100 (12.1)	69.8 (9.2)	61.8 (10.7)

*p<0.05, **p<0.01 compared with Tg2576 mice on control diet by 1-way ANOVAs and *post-hoc* Newman-Keuls tests.

N = 9 mice in the control diet group and 6 in the EVP-0015962 treatment groups.

Concurrent with the reduction in amyloid pathology, reactive astrocytosis, visualized by immunostaining with an antibody against glial fibrillary acidic protein (GFAP), trended lower in the neocortex (F[2,18] = 2.72, p = 0.093; Table 1, Figure 9C-D). By t-test, the 60 mg/kg/day group was significantly lower than the control diet group for percent area of reactive astrocytes in the neocortex (p = 0.038). Activated microglia, immunostained with an antibody against CD11b, trended lower in both the neocortex and hippocampus (neocortex, F[2,18] = 2.57, p = 0.104; hippocampus, F[2,18] = 3.37, p = 0.057; Table 1, Figure 9C-D). By t-test, the reduction in activated microglia reached significance in a comparison of 60 mg/kg/day and control diet groups in the hippocampus (p = 0.046), but was a trend in the neocortex (p = 0.069).

### Contextual fear conditioning deficits are reversed by chronic treatment with EVP-0015962

**Figure 10:**
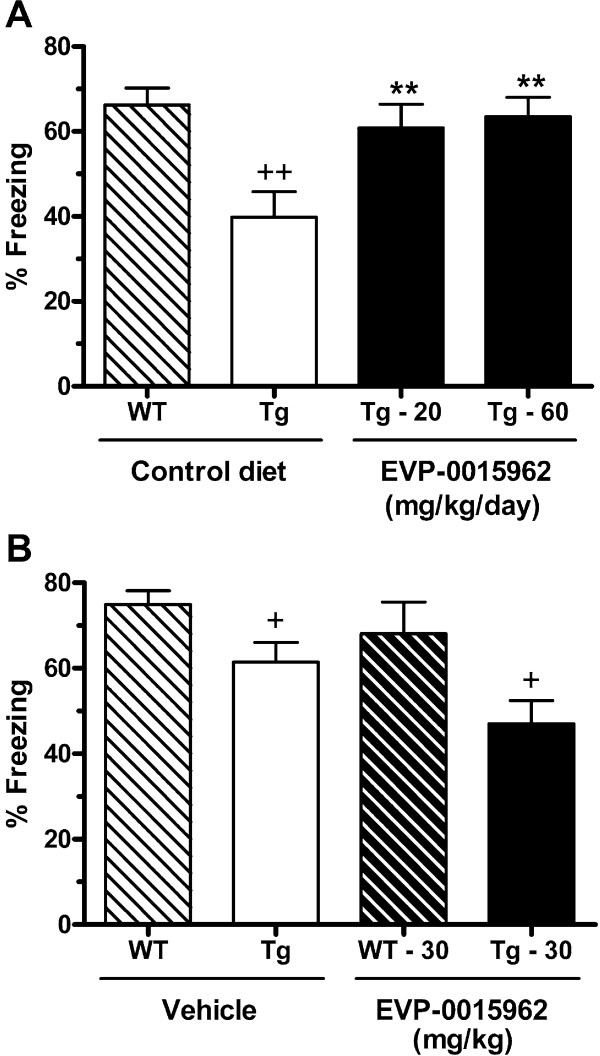
**Chronic daily exposure to EVP-0015962 in Tg2576 mice reverses a cognitive decline. A.** Percent freezing in the CFC behavioral assay after chronic treatment with EVP-0015962 for 11 weeks in wild type (WT) and Tg2576 (Tg) mice, aged 30–33 weeks. Difference between WT and Tg2576 mice on control diet (i.e., genotype effect): ^++^p < 0.01. Differences from Tg2576 mice on control diet (i.e., treatment effect): **p < 0.01. N = 10–15 mice per group. **B.** Percent freezing comparing WT and Tg2576 at 30 weeks of age and treated with a single acute dose of 30 mg/kg, p.o. of EVP-0015962 or vehicle. Differences from WT mice receiving the same treatment, either vehicle or 30 mg/kg of EVP-0015962: ^+^p < 0.05, indicating a genotype effect but no treatment effect. N = 10–15 mice per group. All statistical analyses were performed with 1-way ANOVAs and *post-hoc* Newman-Keuls tests or t-tests.

Contextual fear conditioning (CFC) is an important behavioral assay for studying learning and memory related to hippocampal function [38], and has shown deficits in Tg2576 mice that are reversed by both GSIs and GSMs [39,40]. Beginning at 19–22 weeks of age, Tg2576 mice were treated with EVP-0015962 and Tg2576 and WT mice were treated with control diet for 11 weeks. In the CFC assay there was an overall significant effect on percent freezing behavior (F[3,46] = 5.95, p = 0.002, Figure 10A). In *post hoc* analysis, a cognitive deficit (decrease in percent freezing behavior) was detected at 30–33 weeks of age in the Tg2576 mice on control diet compared to WT mice on control diet (p < 0.01). This genotype-associated deficit in theTg2576 mice was reversed by treatment with 20 or 60 mg/kg/day of EVP-0015962 (p < 0.01).

A satellite group of Tg2576 and WT mice at 30 weeks of age received a single administration of EVP-0015962 (30 mg/kg, p.o.) on day 1, 3 h prior to the training session, in order to evaluate whether there were any acute effects of EVP-0015962 treatment on cognition. Overall, there were group differences in percent freezing (F[3,48] = 5.67, p = 0.002, Figure 10B). There was a significant genotype-associated deficit in the Tg2576 mice compared with WT mice for the vehicle-treated mice and EVP-0015962-treated mice (t-tests, p < 0.05). However, neither the Tg2576 nor WT mice treated with 30 mg/kg, p.o. of EVP-0015962 showed an increase in percent freezing compared to their respective vehicle-treated groups.

## Discussion

The presence of neuroinflammation in AD and the seemingly positive effects of chronic NSAID usage on the reduction of the risk of developing AD in retrospective epidemiological studies [20,41] suggested that NSAIDs might be operating via the inhibition of COX or activation of the peroxisome proliferator-activated receptor γ. Indeed, studies in Tg2576 mice generated evidence to support this hypothesis. Tg2576 mice that were fed a diet of 375 ppm of ibuprofen for 4–6 months, showed a marked decrease in amyloid plaque load, as well as in inflammatory mediators, such as interleukin-1β, GFAP, and markers of microglial activation [42,43]. However, subsequent mechanistic studies suggested that certain NSAIDs were able to produce a preferential reduction of Aβ_42_, without altering the levels of other Aβ peptides, presumably through a direct modulation of γ-secretase [22,23,44]. The result of γ-secretase modulation by NSAIDs was an overall shift in the Aβ_42_/Aβ_40_ ratio, with an accompanying increase in the shorter Aβ_38_, both of which were not correlated to COX activity or the activity of other enzymes inhibited by NSAIDs [22-24].

The first generation GSM to be tested in the clinic was Flurizan™, the *R* enantiomer of the racemate NSAID, flurbiprofen [44,45]. Flurizan™ has the classic GSM signature of lowering Aβ_42_ without affecting the total amount of Aβ peptides. However, Flurizan™ was a relatively weak GSM (IC_50_ > 300 μM for Aβ_42_[46]), with low brain concentrations in animals and man, and was subsequently withdrawn from clinical evaluation due to the lack of efficacy in a large phase III trial [47]. More recently, second generation GSMs from multiple chemical classes have been reported, which have improved potency and brain concentrations [25-29,44,48]. In the current study, we have characterized EVP-0015962, a potent small molecule compound, which has a substantially improved potency (4,500-fold) and pharmacological profile compared with Flurizan™. EVP-0015962 had a measured LogD of 3.88 and was present in the brain at 1.3 to 4.3 μM after single oral doses of 10 and 30 mg/kg, respectively. Moreover, we have demonstrated that γ-secretase activity is modulated by EVP-0015962 to selectively decrease Aβ_42_ levels (IC_50_ = 67 nM) in H4-APP_751_ cells, and increase levels of the shorter Aβ_38_ peptide (EC_50_ = 33 nM), without a change in Aβ_Total_ (composed primarily of Aβ_40_[7]) or in Aβ peptides of other lengths. These changes in the levels of Aβ_42_ and Aβ_38_ peptides were seen in the *in vitro* assay systems, as well as *in vivo* in Tg2576 mice after acute treatment. The lack of overall change in Aβ peptide levels and the increase in Aβ_38_ by EVP-0015962 suggest selective modulation, rather than inhibition of the γ-secretase enzyme complex, similar to the mechanism of action of other GSMs with similar chemotypes [48]. In contrast, GSMs of other chemical classes do reduce Aβ_40_ peptide levels, albeit with a higher IC_50_ than that for Aβ_42_, suggesting that they might function differently than EVP-0015962 [29,48].

We further substantiated the modulatory effects of EVP-0015962 by determining whether the compound inhibited the S3/ε-cleavage activity of the γ-secretase complex at its other natural substrates. EVP-0015962 did not inhibit the ability of γ-secretase to generate AICD and NICD *in vitro*. As expected from a lack of effect on AICD formation, no accumulation of APP α- and β-CTFs was observed in the presence of EVP-0015962 either *in vitro* or *in vivo*. EVP-0015962 also did not cause the accumulation of EphA4 CTF. The lack of effect on the processing of other γ-secretase substrates is thus also consistent with γ-secretase modulation. This suggests that EVP-0015962 may avoid the mechanism-based side effects that have been previously associated with GSIs (e.g., intestinal goblet cell hyperplasia and immunosuppression due to inhibition of Notch receptor processing and cognitive impairment due to the accumulation of APP β-CTF) [16,19]. Furthermore, in our longitudinal efficacy study in Tg2576 mice, in which we observed a significant reduction of Aβ_42_, we found no evidence of effects on intestinal goblet cells or on any other organ system examined in satellite animals treated with up to 60 mg/kg/day of EVP-0015962 for 11 weeks. The tolerability of EVP-0015962 was also demonstrated by the similar weight gains at the 20 and 60 mg/kg/day doses of EVP-0015962, when compared to Tg2576 mice on the control diet for 50 weeks. In addition, when survival was examined after 50 weeks of treatment, 25% of the high (60 mg/kg/day) dose group was lost compared to 27% of the control diet group. The observed mortality rates were consistent with the > 20% rates reported for this transgenic model by the supplier (http://www.taconic.com/wmspage.cfm?parm1=2646).

One of the major goals of the longitudinal study was to determine the effects of EVP-0015962 on AD-like pathology in a transgenic mouse model. The Tg2576 mouse model expresses high levels of APP and Aβ with a transgene containing human APP695 bearing the Swedish mutation (K670N/M671L) behind the hamster prion protein gene promoter [36]. One significant advantage of the Tg2576 model is that diffuse and neuritic plaques begin to appear at approximately 6–7 months and, along with extractable Aβ, increase with age [37]. Furthermore, these age-related changes are coincident with the appearance of cognitive deficits [36,49], suggesting that these mice are a useful tool for studies of Aβ modifying therapies.

In an interim assessment, after 11 weeks of chronic treatment with EVP-0015962, we examined the behavioral effects of GSM treatment. Studies have shown that treatments that lower Aβ can restore cognitive function in these animals [39,40]. In the CFC assay, which is primarily hippocampal dependent [38], the Tg2576 mice displayed the expected cognitive deficit in contextual memory at 30–33 weeks of age. Importantly, the cognitive deficit was reversed and/or prevented at both dose levels following 11 weeks of treatment with EVP-0015962. Separate groups of Tg2576 and WT mice received a single administration of EVP-0015962 (30 mg/kg, p.o. on day 1, 3 h prior to training) in order to evaluate the acute effects of EVP-0015962 treatment on CFC. Overall, differences between the genotypes were observed in this study (i.e., a genotype-associated deficit in the Tg2576 mice). However, acute dosing with EVP-0015962 did not reverse this deficit. This is in contrast to the report that 100 mg/kg, p.o. of the GSI DAPT, administered 3 h before training in Tg2576 mice, lowered Aβ levels and rapidly improved the CFC cognitive deficit [39]. The WT mice, however, did not have improved CFC performance after DAPT treatment, suggesting that the effect was due to Aβ lowering, rather than to a general cognitive enhancing effect, as observed with rolipram in the same study. It is unclear by what mechanism a GSI improved cognition acutely and why the GSM EVP-0015962 did not. In a Y-maze spontaneous alternation test of 5.5-month-old Tg2576 mice, acute GSI treatments also improved short term spatial memory, but 8 days of treatment did not [19]. This lack of effect of subchronic treatment with GSIs on spatial memory was attributed to the accumulation of APP β-CTF in hippocampal synapses and was consistent with findings in the clinic of worsening of cognition and the ability to perform activities of daily living in AD patients [18]. In contrast, GSM-2 improved short term spatial memory after both 1 and 8 days of treatment, suggesting that acute effects of GSMs on cognition can be observed under some experimental conditions [19].

After 50 weeks of chronic daily treatment with EVP-0015962 in a food formulation, which began before amyloid plaque deposition (17–26 weeks of age), brain levels of soluble Aβ_42_ were dose dependently reduced. Additionally, increased levels of Aβ_38_ were observed in the TBS soluble pool of Aβ in the 60 mg/kg/day group. Aβ_Total_ was not changed, similar to what was observed in C57BL/6 mice (data not shown) and Tg2576 mice administered a single dose of EVP-0015962. These findings indicated that EVP-0015962 was modulating Aβ production in the Tg2576 mice in a similar fashion, whether there were Aβ deposits or not. In addition, Aβ peptides, particularly Aβ_42_ and Aβ_Total_ (primarily Aβ_40_), were decreased in the formic acid extractable pool, albeit to different extents. Since Aβ deposits contain all lengths of Aβ peptides but are primarily composed of Aβ_42_ and Aβ_40_[4-7], this finding is consistent with a reduction in Aβ deposition and is supportive of a potential disease modifying effect of EVP-0015962.

As suggested by the reductions in insoluble Aβ peptide levels after chronic treatment with EVP-0015962, Aβ aggregates were also significantly lowered by the high-dose treatment. The degree of reduction in aggregated Aβ (73%) after treatment with 60 mg/kg/day of EVP-0015962 was consistent with the reduced levels of Aβ_42_ detected by enzyme-linked immunosorbent assay (ELISA) after formic acid extraction of insoluble Aβ (86%) and of Total Aβ detected by Western blotting (81%). Interestingly, more disparity among the measures in amount of Aβ reduction was observed for the 20 mg/kg/day treatment. In the aggregated Aβ assay the reduction was 19%, whereas the reductions by ELISA and Western blotting were larger and similar (about 40–50%).

Chronic treatment of Tg2576 mice with other GSMs has demonstrated reductions in amyloid deposition [25-27]. Similarly, the histological analyses of the chronically treated Tg2576 mice demonstrated reductions in percent area and number of amyloid plaques in the neocortex and hippocampus. Although there was only a small non-significant difference between the two doses of EVP-0015962, there was a general trend towards a more pronounced effect at the 60 mg/kg/day dose. Significant reductions were observed in the aggregated Aβ of the plaque cores (visualized with ThioflavinS) and in the more diffuse Aβ surrounding the plaque cores (visualized with the 6E10 antibody) [50-53]. Although both staining procedures produced significant EVP-0015962 treatment effects for percent area and number of plaques, plaque size was only reduced in the ThioflavinS analysis. Since the cores of amyloid plaques are initially seeded by aggregated Aβ_42_[5], and this is the peptide most reduced by EVP-0015962 treatment, it is likely that the higher number of significant findings with ThioflavinS relates to its preferential staining of the plaque cores, while 6E10 antibody recognizes Aβ of various lengths and states of aggregation, which were less altered overall by the treatment. This suggests that EVP-0015962 might mediate its effect through a reduction of the amount of Aβ_42_ available for the initial seeding and growth in the size of amyloid plaque core, and maybe less through the subsequent growth of plaques by the addition of Aβ peptides of other lengths.

The effect on reactive gliosis was pronounced, and may have been the result of the significant reduction in the amyloid plaque load and/or a direct anti-inflammatory activity of EVP-0015962. In enzyme assays for COX-1 and COX-2, the IC_50_ of EVP-0015962 for COX-1 was > 10 μM (n = 2), and the percent inhibition for COX-2 at 30 μM was 56% (n = 2), well above the *in vitro* IC_50_ for Aβ_42_ (67 nM) and the brain exposures of EVP-0015962 after chronic treatment. However, effects of EVP-0015962 on other inflammatory pathways have not been excluded. Nevertheless, since the reactive gliosis in Tg2576 mice on the control diet was localized around the amyloid plaques and Aβ can activate astrocytes and microglia, as well as induce inflammatory responses [20], it is likely that the EVP-0015962-induced decrease in Aβ_42_ and amyloid plaques at least partially led to the attenuated reactive gliosis. These data suggest that a therapy which reduces Aβ_42_ levels will effectively reduce amyloid plaque formation and the concomitant reactive gliosis.

## Conclusions

We have demonstrated that EVP-0015962 behaves as a GSM. The compound was orally bioavailable, detected in brain, well tolerated after daily treatment in Tg2576 mice, and decreased Aβ deposition and reactive gliosis. EVP-0015962 was also shown to prevent and/or reverse hippocampal cognitive deficits associated with the decrease in amyloid deposition after chronic treatment. EVP-0015962 is a novel, small molecule GSM with the potential to be a well-tolerated, disease-modifying therapeutic for Alzheimer’s disease.

## Methods

### EVP-0015962

EVP-0015962 was synthesized and chirally separated, as described by Shapiro and Chesworth [54]. For *in vitro* experiments, EVP-0015962 was dissolved in dimethyl sulfoxide (DMSO, Sigma-Aldrich, St. Louis, MO) as a 30 mM stock solution for dilution into medium. For food formulation studies, EVP-0015962 (20 and 60 mg/kg/day) was milled into standard mouse chow pellets (AIN-93M, Research Diets, Inc., New Brunswick, NJ). Diet doses of EVP-0015962 were calculated based on the desired single daily dose, body weight, and daily food intake. The food formulations of 20 and 60 mg/kg/day were equivalent to 171 and 514 ppm of EVP-0015962 in the diet, respectively.

### COX enzyme assays

COX-1 and COX-2 enzyme assays were performed by Cerep (Redmond, WA, Catalog # 0726 and 0727). Briefly, the assays used human recombinant Sf9 cells and arachidonic acid (4 μM for COX-1 and 2 μM for COX-2) as the substrate. Production of PGE_2_ was detected by enzyme immunoassay.

### Animals

Animals were maintained on 12/12 h light/dark cycle with food available *ad libitum*. All procedures were performed with approval from the Institutional Animal Care and Use Committee and were in accordance with the guidelines in the Guide for the Care and Use of Laboratory Animals from the U.S. Department of Health and Human Services.

### Cell-based assay for measuring secreted Aβ peptides

Human neuroglioma H4 cells (ATCC, Manassas, VA) were transfected with a pcDNA3.1 (Invitrogen, Carlsbad, CA) plasmid expressing human wild type APP_751_ cDNA; and a stable cell line was generated using G418 (Invitrogen) selection [55]. Prior to experimentation, H4-APP_751_ cells were maintained in Dulbecco’s modified Eagle’s high glucose medium with 10% fetal bovine serum, 1% penicillin-streptomycin, 2 mM L-glutamine, and 0.4 mg/mL G418. All culture reagents were from Invitrogen.

Cells were plated at 15,000 cells/well in Costar 96-well plates (Corning, Corning, NY) and placed at 37°C and 5% CO_2_. Six hours after plating, cells were washed three times with Pro293™ chemically defined medium (Lonza, Walkersville, MD) with 1% penicillin-streptomycin and 2 mM L-glutamine, followed by addition of EVP-0015962 (0.003–10 μM, final DMSO concentration of 0.33%). Plates were incubated overnight (16–18 h) and supernatant was removed for quantification of Aβ peptides by sandwich ELISA. Cytotoxicity was evaluated using CellTiter 96® AQueous One Solution Cell Proliferation Assay (MTS Assay, Promega, Madison, WI) according to the manufacturer’s protocol.

### Rat primary neocortical cultures

Primary cultures were established from the neocortex of E17 rat embryos obtained from timed pregnant CD rats (Charles River Laboratories, Wilmington, MA). Following tissue dissection and trituration, the cultures were suspended in Neurobasal™ medium (Invitrogen) supplemented with 10% horse serum (Sigma-Aldrich) and 520 μM L-glutamine [56,57]. Cells were plated at 50,000 cells/well in Costar 96-well poly-d-lysine-coated plates. Following incubation at 37°C and 5% CO_2_ for 4–5 h, the plating medium was exchanged with Neurobasal® medium with 2% B-27® supplement (Invitrogen), 520 μM of L-glutamine, and 1% penicillin-streptomycin. Assays were performed at day eight *in vitro* (DIV8) after replacement of one-half of the medium and addition of EVP-0015962 (0.01–30 μM, final DMSO concentration of 0.1%). Cultures were incubated with compound for 24 h for analysis of Aβ peptides by sandwich ELISA and cytotoxicity by MTS assay.

### Aβ measurements

Aβ peptide levels were quantified by sandwich ELISA. Plates were coated overnight at 4°C with either human anti-Aβ_Total_ (amino acids [aa] 27–37) or rodent anti-Aβ_1-x_ (aa 1–8) rabbit polyclonal antibodies. For detection of Aβ_38_ and Aβ_42_, plates were coated with antibodies specific for the carboxyl-terminal neoepitope of each peptide. Antibodies were from Dr. Pankaj Mehta (Institute for Basic Research in Developmental Disabilities, Staten Island, NY) [58].

Freshly collected samples of cultured cell supernatant or synthetic peptides (American Peptide Co., Sunnyvale, CA or California Peptide Research, Inc., Napa, CA) for standard curves were diluted with media, and loaded into anti-Aβ antibody-coated plates and incubated at 4°C for about 24 h. Aβ peptides were detected with the 4G8 antibody (Aβ, aa 17–24) conjugated to horseradish peroxidase (Covance, Inc., Princeton, NJ), and SureBlue 3,3’,5,5’-tetramethylbenzidine (TMB) peroxidase substrate (KPL, Inc., Gaithersburg, MA). Following addition of TMB stop solution (KPL, Inc.), plates were read for absorbance at 450 nm (SpectraMax M5e Microplate Reader, Molecular Devices, Inc., Sunnyvale, CA). EVP-0015962-treated samples were normalized to samples treated with DMSO alone (no inhibition) and to samples treated with DAPT (5 μM, Sigma-Aldrich). IC_50_ and EC_50_ values were calculated from values reported as percent of DMSO controls using nonlinear regression, based on a sigmoidal dose–response (variable slope) model. All data were analyzed in Prism 4 (GraphPad Software, Inc., San Diego, CA).

### MALDI-TOF mass spectrometry

Immunoprecipitation of carboxyl-terminally truncated Aβ peptides from 4 mL of H4 cell media was conducted using Aβ-specific antibodies coupled to magnetic beads as described elsewhere [59]. The anti-Aβ antibodies 6E10 and 4G8 (Covance, Inc.) were used. After elution of the immune-purified Aβ peptides, the detections were performed on an UltraFlextreme MALDI-TOF/TOF instrument (Bruker Daltonics, Bremen, Germany).

### AICD production

The effects of increasing concentrations of EVP-0015962 on cleavage products of APP were tested in a cell-free system. DAPT was used as a reference positive control for the assay. For each assay condition, a 5-μL microsomal fraction, containing 1% CHAPSO (3-[(3-cholamidopropyl)dimethylammonio]-2-hydroxy-1-propanesulfonate) from murine embryonic fibroblasts (MEF) lacking the Aph-1A, Aph-1B, and Aph-1C genes [60] (triple knock out) and rescued with Aph-1A_L_, was prepared in a reaction mixture of 5 μL substrate (recombinant APP β-CTF 3-Flag-tagged) at 0.8 μM, 0.0125% phosphatidylethanolamine, 0.1%, phosphatidylcholine, 0.05% sphingomyelin, and 8.9 μL PIPES (piperazine-N,N′-bis[−ethanesulfonic acid]) buffer. EVP-0015962 (0.001–100 μM) or DAPT (10 μM) in 0.5 μL of DMSO was added to the reaction mixture and incubated at 37°C for 3 h. For detection of AICD, one-half of the reaction mixture was purified by a methanol-chloroform extraction in order to remove lipids and hydrophobic proteins (e.g., APP β-CTF). This purified fraction was further analyzed by SDS-PAGE and Western blotting. AICD was detected with an ANTI-FLAG® antibody (Sigma-Aldrich). The other half of the reaction mixture was used to analyze the levels of Aβ_1-40_ and Aβ_1-42_ by AlphaLisa (Perkin Elmer, Waltham, MA).

### APP CTF assays

To evaluate the accumulation of APP α- and β-CTFs, H4-APP_751_ cells were treated for 16 h with EVP-0015962 or the GSI LY-411,575 (Alchem Laboratories Corp., Alachua, FL) at 0.001–10 μM. Cells were lysed *in situ* and the resulting lysate cleared of nuclei by centrifugation, as described elsewhere [61]. Samples were fractionated by SDS-PAGE on a 12% NuPage gel (Invitrogen), transferred to a nitrocellulose membrane, and immunoblotted with an anti-APP-CTF antibody (Sigma-Aldrich). Following incubation with an IRDye secondary antibody (Li-Cor Biosciences, Lincoln, NE), detection was performed using an Odyssey scanner (LiCor Biosciences).

For determination of APP α- and β-CTFs in Tg2576 mouse brain, hemisphere samples were thawed in ice-cold homogenization buffer (150 mg of tissue/mL, 50 mM Tris and 150 mM NaCl, pH 7.4) with protease inhibitor cocktail (Mini Complete™, Roche Applied Science, Penzberg, Germany). Homogenized samples were spun at 435,000 *g* for 50 min at 4°C. The supernatant was discarded and the pellet resuspended in the same volume of homogenization buffer containing 0.5% Triton®-X 100, 1% deoxycholate, and 3% SDS. The pellets were triturated with a pipette until resuspended and incubated on a rotator for 1 h at 4°C. The samples were then centrifuged at 435,000 *g* for 50 min at 4°C and the detergent-soluble supernatant was collected. Protein concentration for membrane (detergent soluble) fraction was determined using the BCA method (Pierce, Rockford, IL). Thirty micrograms of total protein were separated on a Tricine 10-20% gel (Invitrogen) and transferred to a nitrocellulose membrane. Blots were probed with an anti-APP-CTF antibody (Sigma-Aldrich) or anti-Aβ antibody (clone 6E10) followed by incubation with IRDye secondary antibodies (Li-Cor Biosciences). Detection was performed using an Odyssey scanner (LiCor Biosciences) and blots quantified using ImageJ software (available at http://rsbweb.nih.gov/ij).

### NICD production

HEK293 cells (700,000 cells per assay condition) were transiently transfected with a NotchΔE, myc-tagged construct to produce a truncated Notch1 substrate, which is constitutively cleaved by γ-secretase (S3 cleavage) [62]. Control transfections were performed with an empty vector. Twenty-four hours after transfection, the cells were treated with EVP-0015962 (0.001–30 μM) or DMSO (control) for 20 h. Lactacystin (10 μM, Sigma-Aldrich) was used to prevent degradation of NICD. Cell lysates were analyzed by SDS-PAGE and Western blotting using a c-myc monoclonal antibody (9E10, Santa Cruz Biotechnology, Santa Cruz, CA) and a cleaved Notch1 antibody (Cell Signaling Technology, Beverly, MA). The amounts of NICD and total Notch were quantified and NICD was expressed as a percentage of total Notch. The DMSO control condition was set at 100%. Cell viability was determined using the CellTiter-Blue® Cell Viability Assay (Promega).

### EphA4 CTF assay

Rat primary neocortical cultures (DIV14) were treated with 0.1–10 μM of EVP-0015962 or LY-411,575 for 24 h. Cells were lysed with RIPA buffer (50 mM Tris–HCl, pH 7.4, 150 mM NaCl, 0.25% deoxycholic acid, 1% NP-40, and 1 mM EDTA) (Upstate, Temecula, CA) containing protease inhibitors and samples centrifuged for 8 min at 16,200 *g* to remove cell debris. Protein concentration of the lysate was determined by the BCA method, and 30 μg of total protein were loaded on a 10% Bis-Tris gel (Invitrogen). EphA4 CTF was visualized using an anti-EphA4-CTF antibody (Abnova, Walnut, CA) and an IRDye800CW® secondary antibody (Rockland Immunochemicals, Inc., Gilbertsville, PA). Detection was performed using an Odyssey scanner (LiCor Biosciences) and blots quantified using ImageJ software (available at http://rsbweb.nih.gov/ij).

### Acute treatment study in Tg2576 mice

EVP-0015962 was prepared in a vehicle of 10% DMSO, 15% Solutol® HS 15 (BASF, Ludwigshafen, Germany), 10% ethyl alcohol, and 65% water. The 10 and 30 mg/kg doses were a solution and a suspension, respectively. Male Tg2576 mice (B6;SJL-Tg(APPSWE)2576Kha, model 1349, Taconic, Hudson, NY) were used at 21 weeks of age [36]. Mice (24–35 g body weight) were administered 10 or 30 mg/kg, p.o. of EVP-0015962 without prior fasting, and euthanized by CO_2_ asphyxiation 4 h post-dosing. After extraction of the brain, olfactory bulbs and hindbrain were removed and the cerebral hemispheres were cut into 4 pieces. Cerebellum was removed for bioanalysis of EVP-0015962 concentrations. All tissue samples were weighed, frozen in liquid nitrogen, and stored at −80°C until analysis.

### Chronic treatment studies in Tg2576 mice

Male C57BL/6 mice (Charles River Laboratories, 10–12 weeks of age, 24–32 g body weight) were group housed (3–5 per cage) and used in pilot studies to establish the concentrations of EVP-0015962 for the food formulations for the chronic treatment studies. Mice were placed on the food formulations for 3 or 7 days and were euthanized, as described above, approximately 1 h after the onset of the light cycle.

Male Tg2576 and WT mice were both derived from crosses of male Tg2576 mice and female B6SJLF1 mice (Taconic) and were singly housed upon arrival. At 16–23 weeks of age, mice were placed on the control diet (AIN-93M), and one week later, were switched to the experimental diets (EVP-0015962 at 20 or 60 mg/kg/day) or maintained on the control diet. Tg2576 mice were maintained on the food formulations containing EVP-0015962 for 11, 28, or 50 weeks, and were sacrificed at 2–4 h after the onset of the light cycle. Some animals were euthanized as described above. For histological studies, a subset of mice were euthanized by CO_2_ asphyxiation and transcardially perfused with cold 0.9% NaCl. The right hemispheres were immersion fixed in fresh 4% paraformaldehyde in 0.1 M phosphate buffer, pH 7.4 for 1 h, transferred to a 15% sucrose cryoprotectant for 24 h at room temperature, and on the next day, frozen in isopentane chilled by dry ice for 1 min. The left cerebral hemisphere was cut into 2 pieces, weighed, and frozen.

### Measurement of EVP-0015962 in brain

Cerebellums were homogenized in a Mini-BeadBeater (Biospec Products, Inc., Bartlesville, OK). Brain samples and calibration standards in brain homogenate were prepared for liquid chromatography-tandem mass spectroscopy (LC-MS/MS) by precipitating proteins with acetonitrile and vacuum filtration in the presence of an internal standard (EVP-0015962-D_6_). EVP-0015962 was resolved by HPLC (Shimadzu, Kyoto, Japan) using a reverse-phase Xterra® C18 column (100 × 2.1 mm i.d.) (Waters Corp., Milford, MA). Following separation, the column effluent was introduced into a hybrid triple quadrupole/linear ion trap mass spectrometer (API 3200 Q-Trap, Applied Biosystems, Foster City, CA), optimized for detection of EVP-0015962 and using multiple reaction monitoring with mass transition of 479.300>296.700. Concentration of EVP-0015962 was measured as nanograms per gram of brain and expressed as micromolar.

### Sequential Aβ extraction from Tg2576 mouse brain

In order to examine both soluble and insoluble pools of Aβ, sequential extraction of Tg2576 mouse brain samples was performed to produce 4 pools of Aβ, essentially as described elsewhere [37]. Briefly, brains were homogenized in 25 mM TBS, pH 7.4 with protease inhibitors (Mini Complete™) to release the soluble Aβ into the resulting supernatants after high speed ultracentrifugation. The above procedure was then repeated with the pellets from the previous extraction step, using 1% Triton®-X 100 in TBS with protease inhibitors, 2% SDS with protease inhibitors, and 68.75% formic acid. The extracted Aβ from each step was subjected to ELISA for human Aβ_42_, Aβ_38_, and Aβ_Total_, as described above.

### Detection of aggregated Aβ in Tg2576 mouse brain

The A4 assay (Amorfix Life Sciences, Toronto, Canada) was employed to quantify levels of aggregated Aβ in Tg2576 mouse brains. Brain samples were homogenized in 10 volumes of 2% NP-40 in phosphate buffered saline (PBS, pH 7.4) containing 1 mM phenylmethanesulfonylfluoride (PMSF) and protease inhibitors (Mini Complete™). The homogenates were further diluted in PBS with 0.05% Tween® 20 and 1% bovine serum albumin to a final concentration that would provide a signal within the linear range of the immunoassay. A sample enrichment protocol, proprietary to Amorfix Life Sciences, was used to specifically isolate oligomeric and aggregated Aβ rather than monomeric Aβ. Following enrichment, samples were eluted and disaggregated to allow detection of now monomeric Aβ [63]. Aβ was detected by immunoassay following incubation at 37°C with europium-fluorescent beads coupled to the mouse monoclonal 4G10 antibody (N-terminal Aβ, aa 1–17) and magnetic beads coupled to the antibodies 1F8 (C-terminal Aβ, aa 3–40) and 2H12 (C-terminal Aβ, aa 3–42). 4G10, 1F8, 2H12 were produced by Amorfix Life Sciences. Following incubations the samples were placed on a magnet to isolate the immune complex. The intensity of the europium fluorescent signal was measured using time resolved fluorescence on each sample in triplicate and was taken as being directly proportional to the concentration of aggregated Aβ in the sample. The limit of detection using this assay was 50 fg per well. The fluorescent signal for each mouse relative to the background noise for the assay was then expressed as percent of the control diet group.

### Histology

The right hemispheres were sectioned at 20 μm in the sagittal plane. From 12 levels, 7 sections were retained and processed. Aβ plaque load was quantified in sections doubly stained by immunohistochemistry with 6E10 antibody (N-terminal human Aβ, aa 1–16) and by ThioflavinS. Astrocytes were immunostained using a primary rabbit polyclonal antibody against GFAP (Dako, Glostrup, Denmark) and a fluorescently labelled secondary Cy2 antibody (Jackson ImmunoResearch Laboratories, West Grove, PA). Activated microglia were immunostained using a rat anti-murine CD11b primary antibody (AbD Serotec, Kidlington, UK) and a fluorescently labelled secondary Cy3 antibody (Jackson ImmunoResearch Laboratories). Astrocytes and microglia were labelled in a double incubation and sections were counterstained with DAPI to visualize cell nuclei.

Hippocampus and neocortex were measured separately, and the percent area of immunoreactivity or ThioflavinS staining per brain region was evaluated using automated image analysis software (Image Pro Plus, v. 6.2, Media Cybernetics, Bethesda, MD). Numbers and sizes of Aβ deposits were also counted automatically. Measurements from 5 sections per mouse were averaged and the individual mouse average was used to calculate the group means.

### Contextual fear conditioning assay

A 2-day CFC paradigm was used, and the effects of acute and chronic dosing of EVP-0015962 were assessed. For the acute study, EVP-0015962 was prepared as a suspension in a vehicle of 4.5% DMSO, 15% Solutol® HS 15, 10% ethyl alcohol, and 70.5% water. On day 1, acutely dosed mice were administered 30 mg/kg, p.o. of EVP-0015962, 3 h prior to training. Animals in the food formulation groups were maintained on the compound formulated diets. The training session on day 1 consisted of the following sequence: 3 min acclimation to the test chamber (Kinder Scientific, Poway, CA), 2 s 1.5 mA foot shock, 2 min wait period, another 2 s 1.5 mA foot shock, followed by a final 1 min wait period. On day 2, testing consisted of returning the animals to the chamber for 5 min and the total time spent freezing was measured by an automated software system (Kinder Scientific). Freezing was defined as the absence of movement (i.e., zero beam breaks) and was measured every second for the 5-min trial duration (maximum freezing score of 300). Percentage of time spent freezing for each mouse was calculated using the formula: (number of seconds with zero beam breaks/300) X 100.

### Statistics

*In vivo* data were expressed and graphed as mean ± SEM. Group differences for Aβ peptide levels, APP processing fragments, histological measures, and behavioral measures were assessed by one-way analyses of variance (ANOVA) followed by Newman-Keuls *post-hoc* testing or by Student’s t-test. Differences in aggregated Aβ were assessed by non-parametric Kruskal-Wallis ANOVA followed by Dunn’s test since the groups did not have equal variances. The level of significance was set at p < 0.05 in all tests.

## Competing interests

EnVivo Pharmaceuticals, Inc. has a financial interest in EVP-0015962 and funded this research. KR, KMF, LH, ZT, FA, WL, SH, MJM, DS, ZY, HH, SN, MW, DC, J-FB, EF, MA, DF, LL, HP, RC, and GK received compensation and stock and/or options as current or former employees of EnVivo Pharmaceuticals, Inc. GS, BD, VV, HZ, and EP received compensation as consultants of EnVivo Pharmaceuticals, Inc. LS was an employee of Amorfix, Inc. and received compensation from EnVivo Pharmaceuticals, Inc. BHP and DH are employees of QPS Austria GmbH and received compensation from EnVivo Pharmaceuticals, Inc.

## Authors’ contributions

KR contributed to the experimental design and interpretation of all studies and wrote the manuscript. RC and GS designed EVP-0015962 and oversaw its synthesis. KMF, LH, ZT, FA, WL, SH, MM, DS, ZY, HH, DC, J-FB, EF, BDS, VV, LS, HZ, EP, BHP, DH, LL, and HP contributed to the design, execution and interpretation of the studies. SN and MW performed the bioanalytical analyses. DF performed statistical analyses and contributed to the preparation of the manuscript. MA, RC, and GK contributed to the experimental design and interpretation of studies. All authors have read and approved the final manuscript.
